# Adverse perinatal outcomes of chronic intervillositis of unknown etiology: an observational retrospective study of 122 cases

**DOI:** 10.1038/s41598-020-69191-9

**Published:** 2020-07-28

**Authors:** Aurélien Mattuizzi, Fanny Sauvestre, Gwenaëlle André, Marion Poingt, Camille Camberlein, Dominique Carles, Fanny Pelluard, Patrick Blanco, Loïc Sentilhes, Estibaliz Lazaro

**Affiliations:** 1 Department of Obstetrics and Gynaecology, Bordeaux University Hospital, Place Amélie Rabat Léon, 33000 Bordeaux, France; 2 Department of Pathology, Bordeaux University Hospital, Bordeaux, France; 3 Department of Obstetrics and Gynaecology, Bordeaux Nord Polyclinic, Bordeaux, France; 4 Department of Immunology, Bordeaux University Hospital, Bordeaux, France; 5 Department of Internal Medicine, Bordeaux University Hospital, Bordeaux, France; 6 CNRS-UMR 5164, ImmunoConcEpT, Bordeaux University, Bordeaux, France

**Keywords:** Outcomes research, Chronic inflammation, Autoinflammatory syndrome, Intrauterine growth, Preterm birth

## Abstract

Our study aimed to assess perinatal outcomes and recurrence rate of Chronic Intervillositis of Unknown Etiology (CIUE). We conducted an observational retrospective study in a tertiary care university hospital in France from January 1, 1997 to July 31, 2018. 122 pregnancies (102 women) with CIUE were included. Cases of the Department of Histopathology placenta database were re-analysed independently by three pathologists specializing in fetal pathology. Diagnosis of CIUE was confirmed according to: (1) the presence of cellular infiltrate in the intervillous space, (2) ~ 80% of the mononuclear cells in the intervillous space positive for CD68, (3) infiltration occupying at least 5% of the intervillous space, and (4) no clinical or histopathological sign of infection. Outcomes of pregnancies with CIUE (miscarriages, stillbirths, terminations of pregnancy, live birth with or without prematurity or fetal growth restriction) and proportion of CIUE recurrence were analysed. The lost pregnancies comprised 17 (13.9%) miscarriages, 17 (13.9%) stillbirths, and 18 (14.8%) terminations of pregnancy. Of the 70 (57.4%) pregnancies that led to a live birth, 38 (54.3%) new-borns were premature and 50 (72.5%) exhibited fetal growth restriction. Among the 102 women, 23 subsequently became pregnant, half of whom (n = 11) developed recurrent CIUE. CIUE was associated with high rates of adverse perinatal outcomes, including pregnancy loss, fetal growth restriction, and preterm birth with a risk of recurrence nearly 50%.

## Introduction

Chronic intervillositis of unknown etiology (CIUE) is a rare placental pathology first described by Labarrere and Mullen in 1987^1^. It is histologically characterized by extensive leukocyte infiltration of the intervillous space, primarily by mononuclear cells of maternal origin^1,2^. The immune cell infiltration is associated with local inflammation of the intervillous space and leads to trophoblast necrosis and fibrin deposition^1, 2^. CIUE affects placental exchange and is associated with adverse perinatal outcomes; e.g., miscarriage and severe fetal growth restriction (FGR), which frequently result in stillbirth or induced prematurity^3^. Pregnant women who experience a first episode of CIUE are at high risk of recurrence in subsequent pregnancies^3^.

Since the first anatomopathological description by Labarrere and Mullen^1^, many terms—such as chronic histiocytic intervillositis, massive chronic intervillositis, and intervillitis—have been used to describe this severe and recurrent disease. Infectious differential diagnosis was later described as associated with this anatomopathological feature^4–7^. Subsequently, the need for a term denoting histologic lesion of the placenta of unknown cause was recognized^8^. The term CIUE, proposed by Parant et al*.* in 2009^9^, implies the absence of a known etiology.

The prevalence of CIUE has been estimated at 8–9.6% in cases of spontaneous abortion^10,11^, versus 0.6–3.2% in second- and third-trimester placentas^9,10^. This suggests that CIUE is frequently misunderstood by perinatologists and is likely underdiagnosed. Most of the available epidemiological data on CIUE is from retrospective studies with small sample sizes^1–3,9–17^.

We evaluated perinatal outcomes of pregnant women with CIUE and recurrence rate. We also explored clinical parameters and obstetrical characteristics of pregnant women with CIUE, and, factors related to the perinatal outcomes and recurrence of CIUE, with a focus on clinical parameters and therapeutic interventions.

## Materials and methods

### Study population

A retrospective chart review of consecutive cases with a histopathologic diagnosis of CIUE from January 1997 to July 2018 at the Fetal and Placental Pathology Department of a tertiary care university hospital was conducted among 20,890 placentas of singleton pregnancies analysed during this period. Clinical indications for sending a placenta to the pathology department for examination was miscarriage, ectopic pregnancy, molar pregnancy suspicion, FGR, stillbirth, preeclampsia, premature birth, intrauterine infection, placental abruption, Benckiser haemorrhage, or poor neonatal status (pH < 7.00 or Apgar score < 7 at 5 min). Pregnant women with a histopathologic diagnosis of CIUE, followed-up in five centres, were included in the analysis. The subsequent pregnancies of women with one or more diagnoses of CIUE were analysed. Placental tissues were obtained from the tissue bank of the department of Histopathology (archives). Institutional Review Board Project #GP-CE 2019/03 was approved on February 15, 2019 by the Ethics Committee of the University Hospital of Bordeaux. Informed consent was obtained from all women. All methods were carried out in accordance with relevant guidelines and regulations. STROBE guidelines^18^ were used for conducting the study.

### Screening criteria

Cases were identified by searching the Department of Histopathology placenta database for the term ‘intervillositis.’ Serial 2-µm-thick sections of formalin-fixed paraffin-embedded potential CIUE tissues, stained with hematoxylin–eosin–saffron (HES) or immunostained for CD68, were re-analysed independently by three pathologists specializing in fetal pathology. Discordant cases were included or excluded according to consensus among the three pathologists. Kappa values for interobserver variability of the diagnosis of CIUE was 0.70.

Diagnosis of CIUE was confirmed according to the criteria of Bos et al*.*^8^: (1) presence of cellular infiltrate in the intervillous space, (2) ~ 80% of the mononuclear cells in the intervillous space positive for CD68, (3) infiltration occupying at least 5% of the intervillous space, and (4) no clinical or histopathological sign of infection.

### Data collection

Clinical and obstetrical data were collected from the patient medical records. Data on the following clinical characteristics were obtained when available: ethnicity, addictions, and medical and obstetrical history. The obstetrical parameters evaluated were as follows: age, body mass index (BMI), parity, method of conception, pathology associated with pregnancy, and need for prenatal hospitalization for > 24 h. The following maternal laboratory parameters were assessed: levels of alkaline phosphatase, antinuclear antibodies, and antiphospholipid (aPL) antibodies. The pregnancy-specific alkaline phosphatase reference ranges were obtained from a prior report^19^. A > 1:250 titer of antinuclear antibodies was considered positive. The aPL antibodies evaluated were as follows: lupus anticoagulant, anti-cardiolipin, and anti-β2 glycoprotein I antibodies. The adverse perinatal outcomes assessed were as follows: miscarriage (defined as loss of pregnancy before 14 WGA : weeks gestational age [WGA]), stillbirth (defined as in utero death at or after 14 WGA), FGR (defined as a weight of birth less than the 10th percentile for gestational age) and termination of pregnancy (TOP) (defined as pregnancy which is interrupted for a medical reason). The neonatal outcomes were as follows: gestational age at birth, sex, weight, and weight percentile for live births according to the AUDIPOG study^20^, or the anthropometric norms for stillbirth or TOP used in our Fetal and Pathology Department^21^. We also collected clinical and obstetrical data on pregnancies subsequent to CIUE. Finally, we compared the clinical parameters, obstetrical characteristics, and perinatal outcomes of women who did and did not develop CIUE during a subsequent pregnancy.

### Statistical analysis

Quantitative variables are presented as means with standard deviation, or as medians with interquartile range, and qualitative variables are presented as percentages. The non-parametric Mann–Whitney test was used for univariate analyses of quantitative variables, and Fisher’s exact test was used for univariate analyses of qualitative variables. Correlations between quantitative variables were evaluated by the Spearman nonparametric test. All tests were bilateral and a p-value < 0.05 was taken to indicate statistical significance. Statistical analyses were performed using Prism software (version 7.0a; GraphPad Software Inc., La Jolla, CA, USA).

## Results

### Characteristics of the subjects

**Figure 1:**
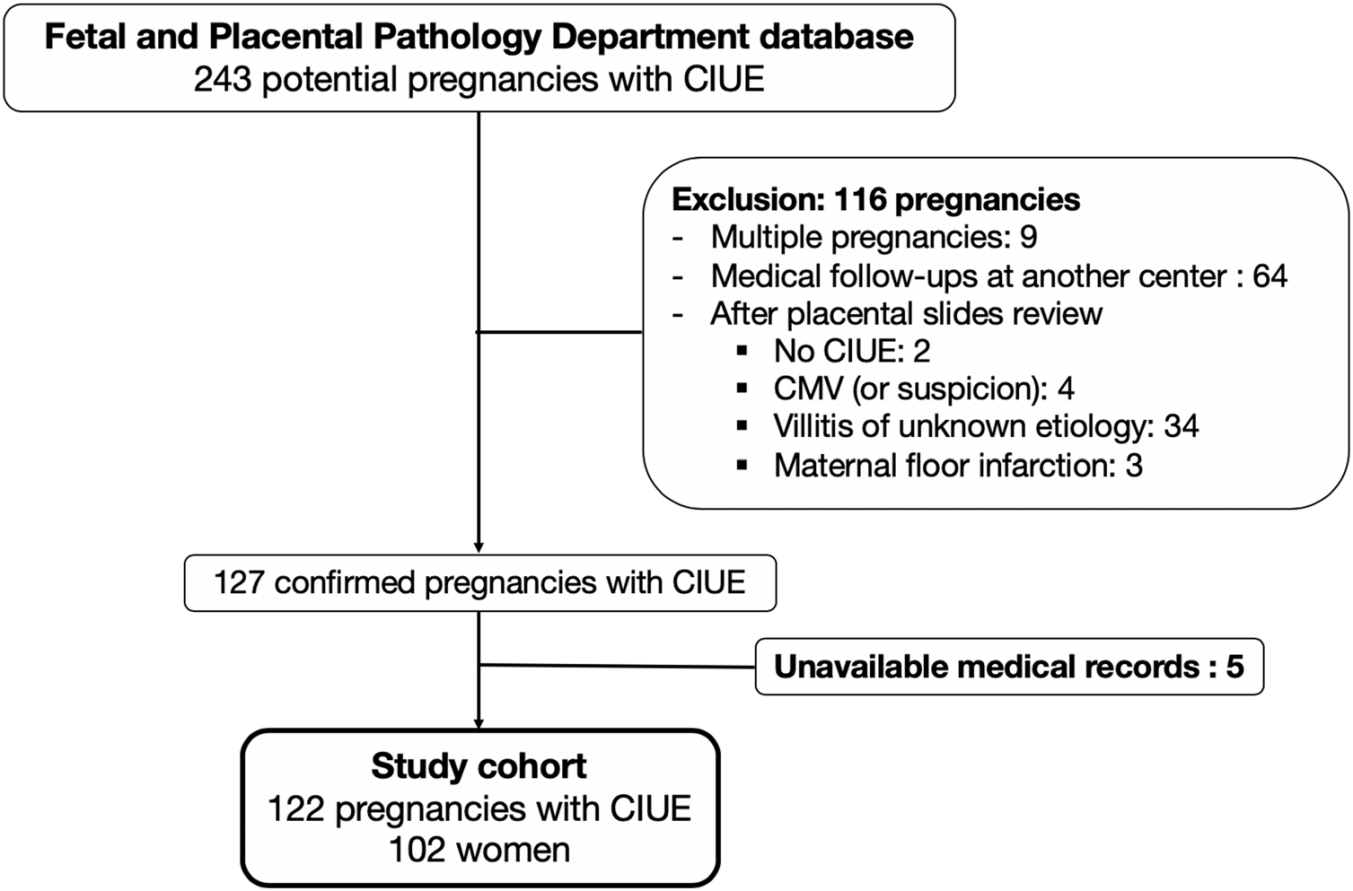
Flow chart of the 122 cases of chronic intervillositis of unknown etiology (CIUE) included in the study.

We identified 243 cases of CIUE in the database of the Fetal and Placental Pathology Department of our institution. A flowchart of the study is shown in Fig. 1. In total, 121 cases were excluded for the following reasons: 9 multiple pregnancies, 64 follow-ups at another centre, 43 without histopathological confirmation of CIUE, and 5 unavailable medical records. A total of 122 of the 243 cases (corresponding to 102 women) met the inclusion criteria.

The clinical and obstetrical characteristics of the subjects are listed in Table 1. Seven women (6.9%) had an autoimmune disease; in four cases, this was diagnosed before their first episode of CIUE, and in the other three after autoimmune investigation triggered by their first episode of CIUE (one case of systemic lupus erythematous associated with aPL syndrome and two cases of obstetric aPL syndrome). Around half of the 79 multigravida women had a history of pregnancy loss; the mean number of prior pregnancy losses was 1.

**Table 1:** Maternal and obstetrical characteristics.

Characteristics	N = 102
**Ethnic group**^**†**^**—no./total no. (%)**	
Caucasian	61/99 (61.6)
Asian	2/99 (2.0)
North African	19/99 (19.2)
South African	15/99 (15.2)
Hispanic	2/99 (2.0)
**Addiction—no. (%)**	
Tobacco	31 (30.4)
Cannabis	3 (2.9)
Other (alcohol, drugs)	0 (0.0)
Chronic hypertension—no. (%)	3 (2.9)
**Diabetes mellitus—no. (%)**	
Type 1	2 (2.0)
Type 2	0 (0.0)
Venous thromboembolic disease—no. (%)	0 (0.0)
**Autoimmune disease—no. (%)**	
Systemic sclerosis	1 (1.0)
Pulmonary sarcoidosis	1 (1.0)
Hashimoto thyroiditis	2 (2.0)
Systemic lupus erythematous + aPL syndrome	1 (1.0)
Obstetrical antiphospholipid syndrome^¥^	2 (2.0)
Congenital thrombophilia—no. (%)	0 (0.0)
**History of pregnancy loss before CIUE**	
< 14 WGA only—no./total no. (%)	36/79 (45.6)
Miscarriage—no	34
Ectopic or molar pregnancy—no	2
≥ 14 WGA only—no./total no. (%)	4/79 (5.1)
Both < 14 WGA and ≥ 14 WGA—no./total no. (%)	4/79 (5.1)

*aPL* antiphospholipid, *CIUE* chronic intervillositis of unknown etiology, *WGA* weeks gestational age.

^†^Data are missing for three women.

^¥^According to the Sapporo criteria^30^. Antibody positivity was confirmed 12 weeks after delivery.

The maternal characteristics and laboratory parameters of the 122 cases of CIUE are listed in Table 2. Almost all of the pregnancies were obtained spontaneously. Among the 93 pregnancies that reached ≥ 22 WGA, prenatal hospitalization was required for 73 (78.5%), mostly due to FGR. Preeclampsia and premature preterm rupture of membranes (PPROM) occurred in 8.6% and 10.8% of cases, respectively. The alkaline phosphatase level was measured in 59 cases that reached ≥ 22 WGA. Using upper limits of 126 and 229 UI/L for the second and third trimesters, respectively^19^, 61% of the 59 cases had an abnormal alkaline phosphatase level. The alkaline phosphatase level was not significantly different between cases of pregnancy loss and live birth (median, 296.5 *vs*. 265 IU/L, respectively, p = 0.44) and was not correlated with the birth weight percentile (p = 0.23, r =  − 0.16).

**Table 2:** Characteristics and perinatal outcomes of pregnancies with CIUE.

Clinical characteristic	N = 122
**Age—year**	
Median [interquartile range]	32 [28–36]
Age ≥ 35 y—no. (%)	37 (30.3)
**BMI at first trimester†**	
Median [interquartile range]	22 [20–25]
BMI ≥ 30—no. (%)	10 (8.2)
Primigravida—no. (%)	23 (18.9)
**Method of conception—no. (%)**	
In vitro fertilization	3 (2.5)
Ovulation induction or artificial insemination	4 (3.3)
Spontaneous	115 (94.2)
**Pregnancy ≥ 22 WGA—no./total no. (%)**	93/122 (76.2)
Gestational diabetes mellitus	10/93 (10.8)
Gestational hypertension	5/93 (5.4)
Preeclampsia	8/93 (8.6)
Preterm labor	9/93 (9.7)
Premature preterm rupture of membranes	10/93 (10.8)
Prenatal hospitalization	73/93 (78.5)
Fetal sex ratio M/F	1.02
**Biological characteristics**	**no./total no. (%)**
Abnormal alkaline phosphatase ≥ 22 WGA^¥^—no./total no. (%)	36/59 (61.0)
Significant antinuclear antibodies^‡^—no./total no. (%)	10/73 (13.7)
Positive antiphospholipid antibodies—no./total no. (%)	6/71 (8.5)
Lupus anticoagulant	3/71 (4.2)
Anti-cardiolipin	1/71 (1.4)
Lupus anticoagulant and anti-β2glycoprotein-I	2/71 (2.8)
**Obstetrical outcomes**	**N = 122**
**Miscarriage—no. (%)**	**17 (13.9)**
Week of gestation—mean ± SD	8 ± 2
Repeated miscarriage^§^—no./total no. (%)	7/17 (41.2)
**Stillbirth**^**†**^—**no. (%)**	**17 (13.9)**
Week of gestation—mean ± SD	22 ± 6
FGR^*£*^—no./total no. (%)	14/15 (93.3)
< 3^rd^ percentile	12/14 (85.6)
**Termination of pregnancy—no. (%)**	**18 (14.8)**
Week of gestation—mean ± SD	24 ± 3
FGR^*£*^—no./total no. (%)	17/17 (100)
< 3rd percentile	16/17 (94.1)
**Live birth—no. (%)**	**70 (57.4)**
FGR^*£*^—no./total no. (%)	50/69 (72.5)
< 3rd percentile	36/50 (72.0)
Prematurity—no./total no. (%)	38/70 (54.3)
< 28 WGA	2/38 (5.3)
[28 – 31^+6^ WGA]	9/38 (23.7)
[32—33^+6^ WGA]	6/38 (15.8)
[34—36^+6^ WGA]	21/38 (55.3)
Neonatal death no./total no. (%)	4/70 (5.7)
Live birth without comorbidity	7/70 (10.0)

*BMI* body mass index, *WGA* weeks gestational age, *FGR* fetal growth restriction.

^†^BMI is the weight in kilograms divided by the square of the height in meters.

^‡^≥ 1/250.

^¥^Alkaline phosphatase level was defined as abnormal when it exceeded 126 and 229 UI/L for the second and third trimesters^19^, respectively.

^§^Three or more consecutive miscarriages.

^†^Only one stillbirth was not associated with an FGR, instead occurring in the presence of uncontrolled diabetes.

^£^Weight data are missing for two stillbirths, one pregnancy termination and one live birth.

### Perinatal outcomes

The perinatal outcomes are listed in Table 2. Miscarriage occurred in 17 (13.9%) of the 122 pregnancies, > 40% of which were recurrent miscarriages. Seventeen pregnancies (13.9%) led to a stillbirth, in 14 cases due to FGR (93.3%; weight data missing for two neonates). One stillbirth was due not to FGR, but rather to uncontrolled diabetes mellitus. Eighteen pregnancies (14.8%) were terminated due to very early FGR. Among the 70 pregnancies that led to a live birth (57.4%), 72.5% exhibited FGR and 54.3% were premature. Four neonates (5.7%) died before day 28 of life. Only 10% of the live births reached 37 WGA without any weight restriction. FGR was reported in 80 of 101 births at > 14 WGA (79.2%; weight data missing for 4 neonates).

### Recurrence of CIUE

The distribution of new pregnancies according to CIUE and recurrent CIUE is shown in Supplementary Fig. 1. In total, 23 of the 102 women (22.5%) had at least one new pregnancy after the first episode of CIUE (total of 40 pregnancies), and 11 women (47.8%) experienced at least one recurrence of CIUE (among 27 pregnancies: 20 of recurrent CIUE, 1 of non-recurring CIUE, and 6 of unknown status; i.e., no analysis of placental histopathology); 9 (39.1%) did not experience recurrence of CIUE (among 10 pregnancies: 10 of non-recurring CIUE), and 3 (17.4%) had three miscarriages (unknown status). The median number of recurrences was one. None of the pregnancies with recurrent CIUE had a different father or resulted from donated gametes.

We compared the maternal characteristics and obstetrical outcomes (Table 3) according to recurrence of CIUE. The pregnant women with recurrent CIUE were older than those without recurrent CIUE (34 *vs*. 28 years, p = 0.003), and had a significantly higher BMI (23.8 *vs*. 22.6 kg/m^2^, p = 0.02). None of the other clinical or maternal characteristics differed significantly according to recurrence of CIUE. The miscarriage rate was significantly higher (51.9% *vs*. 10%, p = 0.03), and the live birth rate was significantly lower (29.6% *vs*. 80.0%, p =0.01), in the recurrent than in the non-recurrent CIUE group. Among the 27 pregnancies of the 11 patients with recurrent CIUE: (1) 20 cases had confirmed recurrent CIUE on pathology with only 15% (3/20) which reached 37 WGA without any weight restriction; (2) 6 pregnancies had unknown status, 5 led to miscarriages and one to a live birth at 38 WGA without weight restriction ; (3) one patient had one pregnancy without CIUE which led to a miscarriage (among 6 new pregnancies after the first episode of CIUE). Recurrent CIUE pregnancies did not have a worse perinatal prognosis than non-recurrent CIUE pregnancies (data not shown).

**Table 3:** Characteristics and outcomes of women with a new pregnancy after CIUE.

Characteristic	Recurrent CIUE N = 11	No recurrent CIUE N = 9	p value*
**Ethnic group**^**†**^**—no**			> 0.99
Caucasian	9	8	
North African	2	1	
**Addiction—no. (%)**			> 0.99
Tobacco	6	4	
Cannabis	–	–	
**Pregnancies characteristics**	**N = 27**	**N = 10**	**p value**
Age—y			**0.003**
Median	34	28	
Interquartile range	33–39	23–33	
Age ≥ 35 y—no. (%)	13 (48.1)	2 (20.0)	
**BMI at first trimester**^**†**^			**0.02**
Median	23.8	22.6	
Interquartile range	22.4–25.6	19.8–25.2	
BMI ≥ 30—no. (%)	1 (3.7)	–	
**Pregnancies ≥ 22 WGA—no./total no. (%)**	11 (40.7)	9 (90.0)	**0.01**
Gestational diabetes	1/11 (9.1)	–	> 0.99
Preeclampsia	–	–	
Premature delivery threat	–	1/9 (11.1)	> 0.99
Premature rupture of membranes	–	1/9 (11.1)	> 0.99
Prenatal hospitalization	8/11 (72.7)	4/9 (44.4)	0.10
**Obstetrical outcomes**			
**Miscarriage—no. (%)**	**14 (51.9)**	**1 (10.0)**	**0.03**
**Stillbirth—no. (%)**	**1 (3.7)**	**1 (10.0)**	0.47
Week of gestation	35	36	
FGR	< 3th Percentile	No	
**TOP—no. (%)**	**4 (14.8)**	**0 (0.0)**	0.56
Week of gestation—mean	23	–	
FGR < 3^rd^ percentile—no./total no	3/4	–	
**Live birth—no. (%)**	**8 (29.6)**	**8 (80.0)**	0.01
FGR—no./total no. (%)	2/7 (28.6) *£*	1/8 (12.5)	0.57
< 3^rd^ percentile	2/7 (28.6) *£*	–	
Prematurity—no./total no. (%)	3/8 (37.5)	1/8 (12.5)	0.57
[28–31^+6^ WGA]	2	–	
[32–33^+6^ WGA]	1	–	
[34–36^+6^ WGA]	–	1	
Neonatal death no./total no. (%)	–	–	
Live birth without comorbidity	4/8 (50.0)	6/8 (75.0)	0.61
**Pathology issue**			
CIUE	20	–	
No CIUE	1	10	
Unknown	6	0	

*CIUE* chronic intervillositis of unknown etiology, *BMI* body mass index, *WGA* weeks gestational age, *FGR* fetal growth restriction, *TOP* termination of pregnancy.

*The non-parametric Mann–Whitney test was used to perform univariate analyses of quantitative variables, and Fisher’s exact test was used for univariate analyses of qualitative variables.

^†^BMI is the weight in kilograms divided by the square of the height in meters.

^£^Weight data were missing for one live birth.

Immunosuppressive, immunomodulatory, and/or thrombolytic therapy was applied in 27 cases after the first episode of CIUE (Supplementary Table 1). The treatments included low-dose aspirin (LDA), alone or in combination with low-molecular-weight heparin (LMWH), steroids, hydroxychloroquine, polyvalent immunoglobulin, or azathioprine. However, none of the treatments impacted the rate of adverse outcomes of pregnancy. Women with more than one recurrence of CIUE frequently underwent treatment with combinations of the above agents, but these had no beneficial effect (Supplementary Fig. 2).

## Discussion

### Main findings

We herein describe the largest case series of CIUE to date. The results confirm that CIUE is associated with high rates of adverse perinatal outcomes. Almost half of the 122 CIUE cases resulted in adverse outcomes, namely miscarriage (13.9%), stillbirth (13.9%), or TOP (14.8%). Over half of the live births were premature and 79.2% had FGR at > 14 WGA.

Our results are consistent with previous reports of the perinatal outcomes of CIUE that analysed relatively few cases (range: 6–69 cases)^1–3,9–15,17,24^. The overall incidence of live births was 30–81%, among which 31–88% were premature and 63–83% showed FGR^9,10,14,17,24^. Marchaudon et al*.*^11^ reported severe FGR (less than the third percentile) in 61.5% of 69 cases of CIUE, which is in accordance with our rate of 63.4%.

In this study, none of the clinical and biological factors investigated—including autoimmune diseases and the alkaline phosphatase level—were associated with adverse perinatal outcomes.

CIUE is reportedly associated with systemic lupus erythematous^11,16^, aPL syndrome^16,25,26^, Sjögren syndrom^27^, and Hashimoto thyroiditis^11,16^. In our study, few women (< 7%) had an autoimmune or inflammatory disease and only a small proportion were positive for autoimmune markers. Marchaudon et al*.*^11^ were the first to report the potential of the alkaline phosphatase level as a marker of CIUE, based on a high alkaline phosphatase level in more than half of the cases showing fibrin deposition in the placenta. Excessive release of alkaline phosphatase could be the result of immune aggression of the syncytiotrophoblast. In our study, the alkaline phosphatase level at > 22 WGA was above the pregnancy-specific normal reference range^19^ in one third of the cases, and was not correlated with birth weight or adverse perinatal outcomes.

Due to a dearth of prospective studies, the recurrence rate of CIUE is unclear. In our study, the rate of recurrent CIUE was 47.8% (11/23), which could be an overestimate as it was based only on new pregnancies monitored in participating centres. A previous retrospective study reported a recurrence rate of 18–67%^10,11^, and a recent systematic review reported a rate of 80%^28^. The only prospective study published to date reported a recurrence rate of almost 30%^16^.

Because of the high risk of recurrence, identifying the optimum preventive treatment is problematic. A variety of therapeutic interventions using various dosages and combinations of treatments have been trialed, but few positive effects have been reported ^3,9,16,24,29^. Indeed, the recent meta-analysis by Contro et al*.* indicated that no currently available treatment improves perinatal outcomes^28^. In our study, various combinations of therapeutic agents (LDA, steroids, hydroxychloroquine, polyvalent immunoglobulin, and azathioprine) failed to improve perinatal outcomes or the recurrence rate. However, the most frequently and intensely treated women also had the most severe recurrent CIUE.

### Strengths and limitations

The strength of our study lies in is its description of 122 pregnancies with CIUE involving 102 women, making it the largest series to date. The women were recruited from primary, secondary, and tertiary care centres, which may have prevented or reduced recruitment bias. Moreover, the placental samples were selected based on the definition of CIUE of Bos et al.^8^, which reduced the heterogeneity of the population.

This study has several limitations. First, there was a potential for selection bias, because this was not a cohort study and systematic analysis of all pregnancies in our centre was not performed; only cases with adverse perinatal outcomes or a history of CIUE were analysed, which may have resulted in overestimation of the incidence of adverse perinatal outcomes. Second, the retrospective design of the study, and the lack of information for some cases, may also have led to overestimation of the incidence of adverse perinatal outcomes and the rate of recurrence. Third, the low statistical power likely hampered identification of prognostic factors for recurrent CIUE.

### Interpretation

This study highlights to perinatologists that CIUE is potentially associated with adverse perinatal outcomes and must be systematically investigated by placental pathological analysis in case of repeated miscarriages, stillbirth or FGR. The risk of recurrence requires close ultrasonography monitoring of fetal growth in case of a subsequent pregnancy.

In the absence of knowledge of the pathophysiology of the disease and controlled trials, identifying the optimum treatment regimen for CIUE is challenging. Fundamental research, including large multicentre epidemiological studies and prospective randomized controlled trials of novel treatments, is needed to improve our understanding of CIUE and the care of the women who suffer with this condition. Transcriptomic or proteomic analysis of placenta tissue could identify the immunological pathway involved in CIUE and lead to the evaluation of specific immunotherapies targeting the relevant immunological pathways.

## Conclusions

CIUE is frequently associated with adverse perinatal outcomes. However, although this is the largest CIUE case series to date, we were unable to identify related clinical and biological factors. The lack of information on the pathophysiology and the factors associated with CIUE hampers patient care. Thus, fundamental research, including large multicentre epidemiological studies and prospective randomized controlled trials of novel treatments, is needed to improve our understanding of CIUE and the care of the women who suffer with this condition.

## Supplementary information

Supplementary Figure 1

Supplementary Figure 2

Supplementary Table 1

## Abbreviations

aPL: Antiphospholipid
AZA: Azathioprine
BMI: Body mass index
CIUE: Chronic intervillositis of unknown etiology
HCQ: Hydroxychloroquine
HES: Hematoxylin–eosin–saffron
FGR: Fetal growth restriction
LDA: Low-dose aspirin
LMWH: Low-molecular-weight heparin
TOP: Termination of pregnancy
MVM: Maternal vascular malperfusion of the placental bed
PIg: Polyvalent immunoglobulin
PPROM: Premature preterm rupture of membranes
WGA: Weeks gestational age
