# Supplementary figures and images for: Adverse perinatal outcomes of chronic intervillositis of unknown etiology: an observational retrospective study of 122 cases
Source: Sci Rep. 2020 Jul 28;10:12611. doi: 10.1038/s41598-020-69191-9 (PMC7387519; doi:10.1038/s41598-020-69191-9)

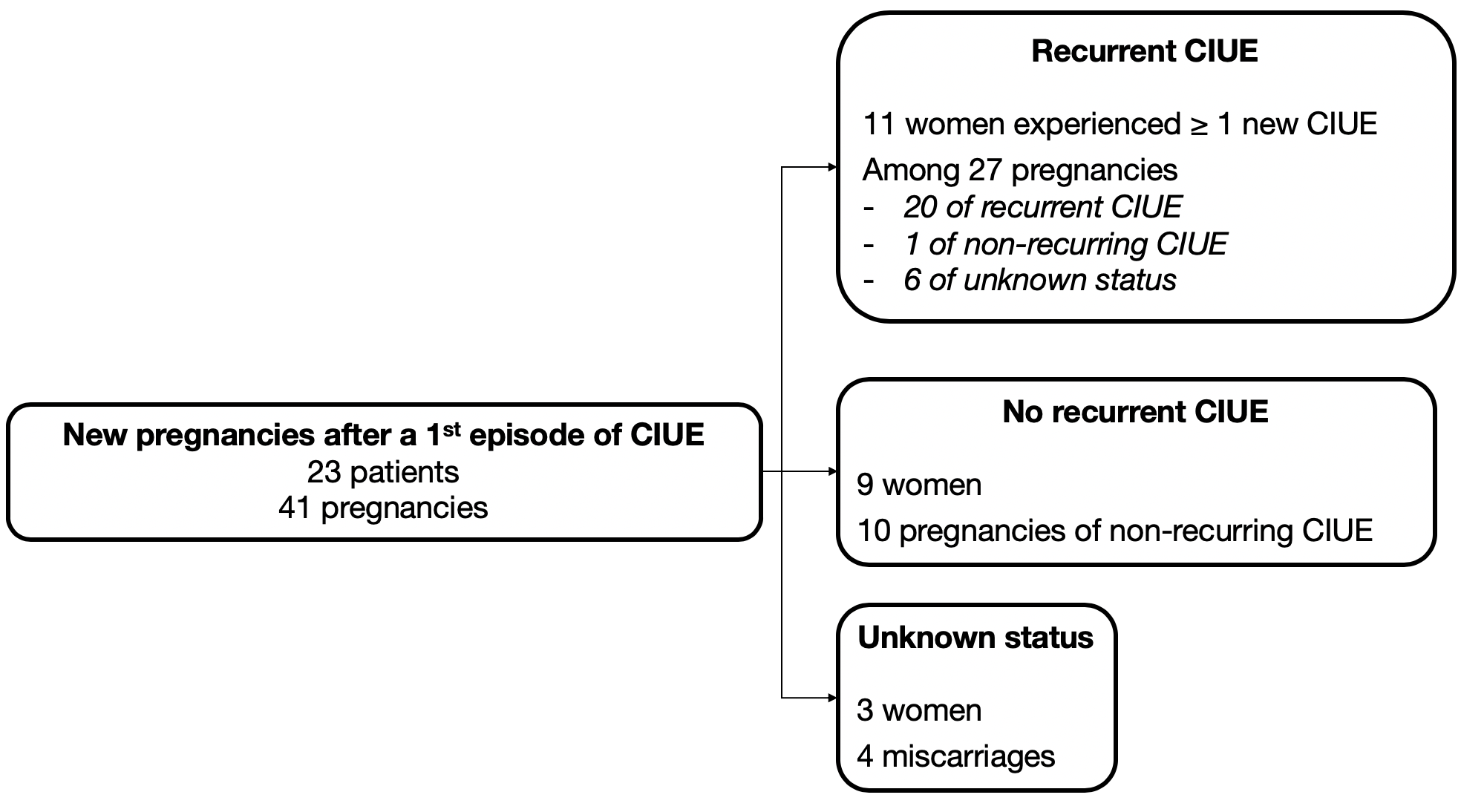

Supplement: Supplementary file 1 — Supplementary Figure 1 [file 41598_2020_69191_MOESM1_ESM.tiff]

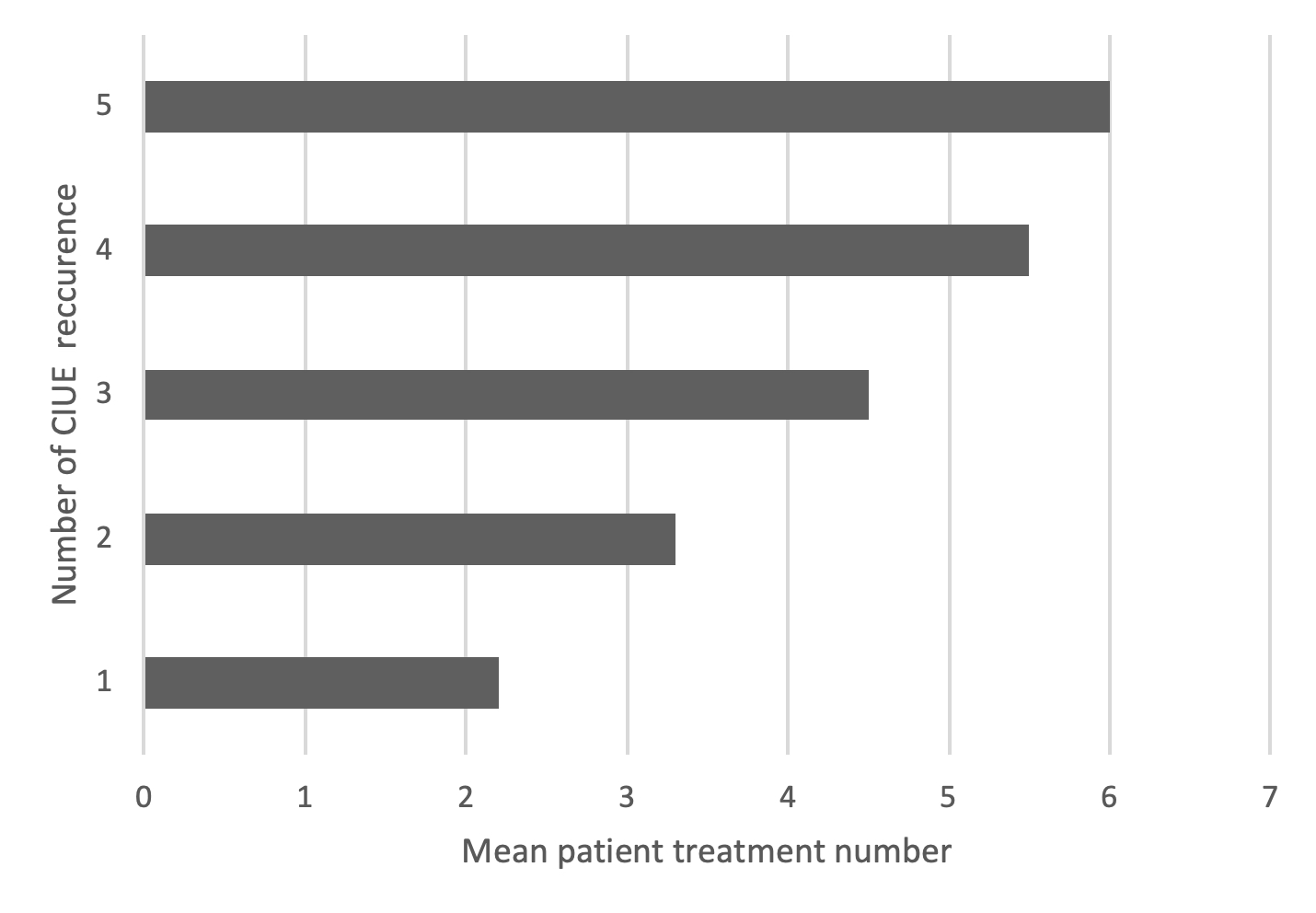

Supplement: Supplementary file 2 — Supplementary Figure 2 [file 41598_2020_69191_MOESM2_ESM.jpg]
