# Supplementary material for: Adverse perinatal outcomes of chronic intervillositis of unknown etiology: an observational retrospective study of 122 cases
Source: Sci Rep. 2020 Jul 28;10:12611. doi: 10.1038/s41598-020-69191-9 (PMC7387519; doi:10.1038/s41598-020-69191-9)
Supplement: Supplementary file 3 — Supplementary Table 1 [file 41598_2020_69191_MOESM3_ESM.docx]

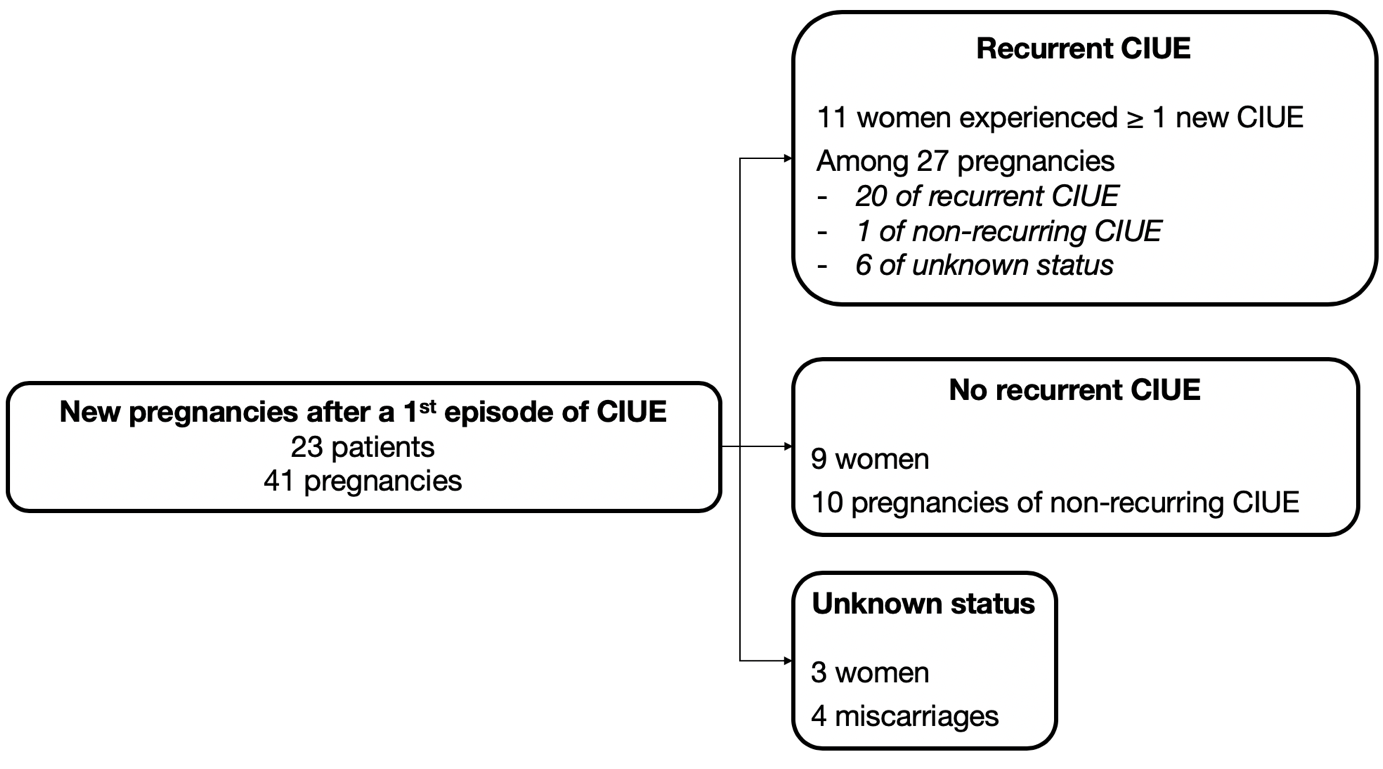
**Supplementary Figure 1.** Distribution of the women with a new pregnancy after CIUE.


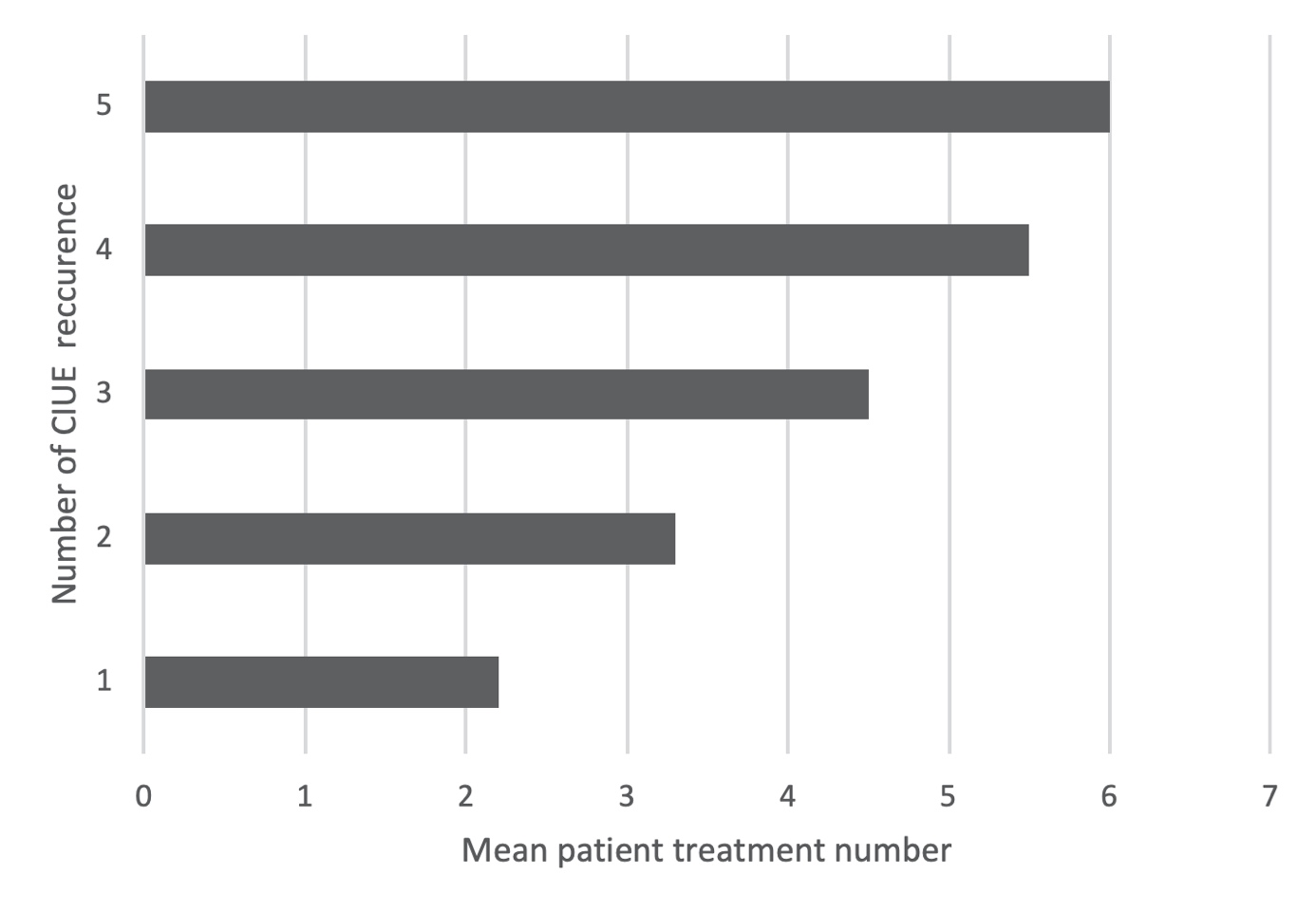
**Supplementary Figure 2.** Mean number of women treated according to the number of CIUE recurrences.

**Supplementary Table 1.** Therapeutic interventions used to prevent recurrence of CIUE according to pregnancy issue.

|  |  | CIUE  N = 19 *†* | | No CIUE  N = 11 *§* | |
| --- | --- | --- | --- | --- | --- |
| Treatment | Total  no.  N = 30*^†^* | Pregnancy loss  no.  N = 12 | Live birth no.  N = 7 | Pregnancy loss  no.  N = 3 | Live birth no.  N = 8 |
| None | 3 | 0 | 1 | 0 | 2 |
| LDA alone | 7 | 4 | 1 | 0 | 2 |
| LDA + LMHW | 5 | 1 | 1 | 0 | 3 |
| LDA + Steroids | 1 | - | - | 1 | - |
| LDA + Steroids + HCQ | 1 | - | - | - | 1 |
| LDA + LMWH + Steroids | 2 | 1 | 1 | - | - |
| LDA + LMWH + Steroids + HCQ | 4 | 2 | 0 | 2 | - |
| LDA + LMWH + Steroids + PIg | 2 | 1 | 1 | - | - |
| LDA + LMWH + Steroids + HCQ + PIg | 3 | 2 | 1 | - | - |
| LDA + LMWH + Steroids + HCQ + PIg + AZA | 2 | 1 | 1 | - | - |

CIUE, chronic intervillositis of unknown etiology; LDA, low-dose aspirin (per os, 100–160 mg per day); LMWH, low-molecular-weight heparin; Steroids, prednisone or prednisolone (per os, 5 to 20 mg per day); HCQ, hydroxychloroquine (per os, 400 mg per day); PIg, polyvalent immunoglobulin (one injection per month); AZA, Azathioprin

† Data were missing for one pregnancy.

§ 10 pregnancies in the recurrence group and 1 pregnancy in the no-recurrence group.
